# Case of Complete Vertical Dislocation of the Patilla

**Published:** 1883-04

**Authors:** Charles T. Parkes


### Article V.

### Case of Complete Vertical Dislocation of the Patella. By Charles T. Parkes, m.d.

In September, 1882, Dr. C. W. Johnson requested me to see a patient who had been injured about the knee some two hours previous to his call. The doctor had already diagnosed a vertical dislocation of the patella. Upon reaching the patient the following history was elucidated. The man, in attempting to get on to his coal wagon, of which he was driver, made a mis-step and fell to the ground with his knee in the bent position. In falling, he struck his right knee against a large piece of coal. Immediately he found he was unable to bend or extend his limb and any attempt to do so was accompanied with terrible pain. Dr. Johnson was called, examined the man, and had him sent to his home.

We found the patient in much pain, with the leg very slightly flexed and fixed in that position. The knee-joint was much broadened anteriorly and the trochlear surface of the lower end of the femur was easily mapped out beneath the stretched integument. To its outer side was a very prominent elevation made of the patella lying in a vertical position and resting upon its inner edge, so that its subcutaneous surface was turned inwards and its articular surface outwards, both of which could be traced beneath the integument.

Diagnosis, a complete outward vertical dislocation of the patella—a half revolution on its longitudinal axis with the inner edge turned downwards and outwards.

The bone was rigidly fixed in its abnormal situation, allowing of no change in its position ; the obstacle to motion consisting in the tense condition of the fascia lata and ligamentum patellæ. The patient was anæsthetized and the reduction attempted. Dr. Johnson flexed the thigh upon the abdomen and extended the leg, both positions being carried to the fullest extreme. I then attempted, by lateral pressure in the opposite direction upon the edges of the patella, to replace it. Several efforts ended in failure, the bone being still held fast by its fibrous attachments. By flexing the leg strongly, I found that the now anterior edge of the bone was drawn outwards in the direction of its normal situation, and the vertical position of the bone altered somewhat. I then directed Dr. Johnson to flex the leg as strongly as he could, and to be ready to extend it suddenly as soon as I gave the word.

After flexion was as complete as it could be made, I placed the end of my pocket knife against the inner (now lower) edge of the bone, pushing against it inwards and forwards, so as to increase the outward inclination of the bone produced by the flexion, and at the same time to raise this edge above the outer rim of the trochlear facet of the femur. As soon as I felt this object was accomplished, sudden extension of the leg was made and the displaced bone slid quietly into its proper position. Hot fomentations were directed to be applied to the joint constantly. The young man remained in bed four days and returned to his work inside of two weeks. There was no troublesome effusion, inflammation or tenderness of the joint following the accident. The patient was a man 20 years old, very tall for his age, rather slender and had large lower joints.

REMARKS.

Vertical dislocations of the patella are acknowledged by all writers to be of rare and unusual occurrence; indeed the time has not long passed since it was considered, by noted surgical men and authors, to be an impossible accident.

Of the twenty or more cases I have been enabled to refer to, none of them are described as being as complete as the one coming under my care. In the recorded cases, the edge of the patella rested in the depression separating the facets of the trochlear surface of the femur, and this position is given as the normal situation of the bone in vertical dislocations by Professor Agnew in his "Surgery," p. 109, Vol. II. In the case now reported, the inner edge of the patella was thrown entirely external, clearing the femoral articular surface absolutely, and rested against the outer tuberosity of the external condyle of the femur, so that the *inner* border of the displaced bone was fully half an inch behind the highest point of the outer rim of the trochlear facet of the femur. Except during voluntary movements of the limb or during passive efforts of flexion or extension, there was no unusual contraction of the quadriceps extensor muscle, it was flaccid and slightly movable laterally. The ligamentum patellæ was very tense, and so also in a remarkable degree was the ileo-tibial band of the fascia lata throughout its entire length. Certainly nothing would have been added to the ease of reduction by a division of the quadriceps tendon—a procedure resorted to in some of the failures to reduce by manipulation. I am convinced that complete anæsthesia banishes all obstacles to a reduction of the displacement, mainly because it does away with the powerful involuntary muscular contractions which accompany the excruciating pain attending even the slightest movements with the bone in its abnormal position. Prolonged or harsh or even mild efforts at reduction without its use are unwarrantable, not only because the coincident muscular spasm render them useless to accomplish any other result than agony to the sufferer, but because such efforts may produce a rent in the fibrous casule of the bone and joint or increase the size of such opening already made by the accident, thus enhancing the liability to effusion into the joint or to dangerous inflammation thereof. Furthermore, the patella may be forced into the opening to such a degree, and held by it so firmly as to make the reduction next to impossible, a condition supposed to have been present in reported cases, which have been left unreduced after many efforts to accomplish it had ended in failure.

The patient should be moved carefully, should be cautioned against any movements whatever of the injured limb ; the surgeon should avoid handling the limb beyond the touch and glance which makes the diagnosis, until the patient is fully anæsthetized.

The contour of the bony surfaces concerned in the accident, the normal looseness of the soft parts surrounding and attached to the patella, especially in the extended position of the leg, are such as to make it impossible for anything in or about them to become an insurmountable obstacle to reduction ; so that I feel justified in saying such difficulties, when present, arise from muscular spasms and the possible changes in the fibrous investments of the bone, perhaps due to the injury—oftener perhaps dependent upon rough and unnecessary manipulations.

